# Rehydration Process in Rustyback Fern (*Asplenium ceterach* L.): Profiling of Volatile Organic Compounds

**DOI:** 10.3390/biology10070574

**Published:** 2021-06-23

**Authors:** Suzana Živković, Marijana Skorić, Mihailo Ristić, Biljana Filipović, Milica Milutinović, Mirjana Perišić, Nevena Puač

**Affiliations:** 1 Institute for Biological Research “Siniša Stanković”—National Institute of the Republic of Serbia, University of Belgrade, Bulevar despota Stefana 142, 11060 Belgrade, Serbia; biljana.nikolic@ibiss.bg.ac.rs (B.F.); milica.milutinovic@ibiss.bg.ac.rs (M.M.); 2 Institute for Medicinal Plant Research “Dr Josif Pančić”, Tadeuša Košćuška 1, 11000 Belgrade, Serbia; mristic@mocbilja.rs; 3 Institute of Physics, University of Belgrade, Pregrevica 118, 11080 Belgrade, Serbia; mirjana@ipb.ac.rs (M.P.); nevena@ipb.ac.rs (N.P.)

**Keywords:** *Asplenium ceterach*, rehydration, volatile organic compounds, lipid peroxidation

## Abstract

**Simple Summary:**

Severe environmental changes, such as drought, can delay growth, the development of plants, and induce injury to their tissues. However, a group of land plant species, called resurrection or desiccation-tolerant plants, is able to lose 95% of their cellular water and still remain viable for long periods, resuming full metabolic activity upon rehydration. Recovery from near-complete water loss is complex and requires the coordination of physical and chemical processes in the resurrection plants. Under stress conditions plants also synthesize and release a wide variety of volatile organic compounds with diverse biological and ecological functions. The rehydration process in resurrection rustyback fern (*Asplenium ceterach*) resulted in complete plant recovery within 72 h, accompanied by high emission of volatiles, mainly belonging to the group of fatty acid derivatives. These findings could have significant implications from biotechnological and ecological perspectives since the rustyback fern has been recently recognized as a valuable source of bioactive compounds.

**Abstract:**

When exposed to stressful conditions, plants produce numerous volatile organic compounds (VOCs) that have different biological and environmental functions. VOCs emitted during the rehydration process by the fronds of desiccation tolerant fern *Asplenium ceterach* L. were investigated. Headspace GC–MS analysis revealed that the volatiles profile of rustyback fern is mainly composed of fatty acid derivatives: isomeric heptadienals (over 25%) and decadienals (over 20%), other linear aldehydes, alcohols, and related compounds. Aerial parts of the rustyback fern do not contain monoterpene-type, sesquiterpene-type, and diterpene-type hydrocarbons or corresponding terpenoids. Online detection of VOCs using proton-transfer reaction mass spectrometry (PTR–MS) showed a significant increase in emission intensity of dominant volatiles during the first hours of the rehydration process. Twelve hours after re-watering, emission of detected volatiles had returned to the basal levels that corresponded to hydrated plants. During the early phase of rehydration malondialdehyde (MDA) content in fronds, as an indicator of membrane damage, decreased rapidly which implies that lipoxygenase activity is not stimulated during the recovery process of rustyback fern.

## 1. Introduction

Certain plant species, termed desiccation tolerant or resurrection plants, have evolved the remarkable ability to withstand extreme dehydration (to just 10% of their water content or less) and resume normal metabolic and physiological activity after rehydration of vegetative tissues without cell damage. Such rapid and ecologically beneficial changes allow a plant to survive equilibrium with 0% air humidity until water becomes available. Upon re-watering, dried resurrection plants quickly revive and become fully photosynthetically active within 24 h [1]. Furthermore, drying and rehydration processes cause only limited damage to resurrection plant tissues, due to a number of morphological, physiological, biochemical and genetically different mechanisms, developed not only for diminishing damages suffered during severe water loss, but also during rehydration [2]. Controlled regulation of physical and metabolic processes enables the minimization of the stress associated with desiccation and allows full recovery once the plant is rehydrated. These adaptations apparently separate resurrection plants from desiccation-sensitive plant species [3]. The desiccated state is correlated with multiple obstacles at the cellular level, such as photo-oxidative stress caused by reactive oxygen species, the metabolic requirements of resurrection, and the mechanical stress of cell and tissue deformation [4,5].

Under stress conditions plants synthesize and release a wide variety of volatile organic compounds (VOCs) with diverse biological and ecological functions. Plant volatiles are usually complex mixtures of diverse organic compounds, including saturated and unsaturated hydrocarbons, esters, aldehydes, ketones, amines, oxides and sulfur compounds, derived from various biochemical pathways [6]. Fatty acid derivatives, including C_6_ green leaf volatiles and their esters, may derive from enzymatic or non-enzymatic reactions [7]. The first response of plants to any environmental changes is closely related to cell membrane structures. Fast and nonspecific response of the membrane is based on the transformation of the cell membrane structural components to signaling compounds. Polyunsaturated fatty acids (PUFAs) are incorporated in cell membranes and together with enzyme lipid peroxidase (LOX) through a series of chemical reactions that give rise to a great variety of products which represents the “nonspecific biological signals” and do not require preceding activation of genes. They are produced as responses to environmental stresses and/or stimuli without requiring any specific gene expression, or long downstream signaling cascades to evoke them [8,9]. Maintenance of membrane integrity is of critical importance to ensure survival upon cellular dehydration. Desiccation stress can result in lipid destruction and membrane damage due to free radical production. It is well known that products of lipid peroxidation (LP) such as malondialdehyde (MDA) are often used as a marker for oxidative stress in plants [10]. The majority of studies on resurrection plants are focused on their strategies to cope with desiccation damage during dehydration, and less attention has been given to the process of rehydration, although the mechanisms for preventing and/or repairing cell damage upon rehydration are of great importance for the desiccation tolerance of resurrection plants [11].

Rustyback fern (*Asplenium ceterach* L.) belongs to the resurrection species and is widespread in Western and Central Europe, including the Mediterranean region. The adult fern (sporophyte) is a perennial herbaceous plant with leathery fronds (species name originates from the dark brown and densely scale-covered lower surface of fronds), which grows in limestone rock crevices and stone walls and may survive long dry periods between wet spells, passing quickly from anabiosis to full biological activity. Although *A. ceterach* belongs to a group of poikilohydric fernsand could sustain different rates of desiccation, recovering uninjured from complete dryness [12], this desiccation tolerant plant still prefers shaded sites with excellent drainageand suitable humid conditions (especially high air humidity) in its habitat, while becoming quiescent when water is unavailable.

Detailed phytochemical analysis of *A. ceterach* and other species from the family Aspleniaceae have been performed recently [13,14,15,16,17]. Froissard et al. [13] reported lipid derivatives as important volatile compounds in *A. ceterach*. The experiments presented here were aimed to study VOCs emission from the rustyback fern sporophyte following transition from dormant (desiccated) to an active, rehydrated state. This was achieved by using coupled Headspace GC–MS and online PTR–MS analysis of the VOCs. Since the first response of plants to any environmental changes is closely related to cell membrane structures, we have also postulated that the rehydration process in *A. ceterach* would disrupt maintenance of membrane integrity. Therefore, we have measured the changes in the lipid status of the fern fronds during rehydration as evidence for the involvement of LOX in the plant cell membrane damage and/or formation of VOCs.

## 2. Materials and Methods

### 2.1. Plant Material

Dormant (desiccated) mature sporophytes of rustyback fern (*Asplenium ceterach* L.) were collected in East Serbia near the Monastery Gornjak (44°15′52.66′′ N 21°32′40.21′′ E) and stored in paper bags at room temperature until use. Species was authenticated by the authors and the corresponding voucher specimens have been deposited at the Department of Plant Physiology, Institute for Biological Research “Siniša Stanković”—National Institute of the Republic of Serbia, University of Belgrade, Serbia.

### 2.2. Headspace GC–MS Analysis of Volatiles

Headspace GC–MS technique was used to identify and quantify volatile organic compounds (VOCs) present in the injected headspace sample. Rustyback fern fronds (dry and fresh) were placed in a closed sampling vessel and deionized water was added. Samples were heated at 80 °C and the vapor in the vessel was sampled for analysis by using a heated gas-tight syringe (Agilent Technologies, Santa Clara, CA, USA). The GC–MS analyses were performed on a Hewlett Packard G1800C-GCD Series II apparatus equipped with a HP-5MS column (30 m × 0.25 mm, 0.25 μm film thickness). Carrier gas was helium (1 mL·min^−1^) and the transfer line was heated to 260 °C. The mass spectra were acquired in EI mode (70 eV) in the *m/z* range of 40–400. Identification of individual components was accomplished by comparison of retention times with standard substances and by matching mass spectral data with those held in the Wiley 275 and NIST libraries of mass spectra. Confirmation was performed by using AMDIS software (AMDIS ver.2.1., National Institute of Standards and Technology-NIST, Standard Reference Data Program, Gaithersburg, MD, USA) and the available literature data [18].

### 2.3. Online PTR–MS Measurements of Volatiles

Desiccated rustyback fern sporophytes similar in size were selected and placed into 350 mL glass jars two hours prior to the experiment and allowed to adapt. Rehydration was initiated by adding 50 mL of deionized water into each jar. Measurements of the ambient level of the VOCs in the atmosphere of glass vessels during the rehydration process were conducted continuously during the next 24 h by using Standard Proton Transfer Reaction Quadrupole Mass Spectrometer (PTR–MS, IoniconAnalytik, GmbH, Innsbruck, Austria). The identification and quantification of VOCs by PTR–MS was based on protonated parent ion masses (molecular mass plus one atomic mass unit), yielded in proton transfer reaction with H_3_O^+^ ions, and the relative abundance of ions was obtained by the use of quadrupole mass spectrometer and secondary electron multiplier. Detailed descriptions of the PTR–MS technology can be found elsewhere [19,20]. In this investigation masses in the range from *m/z* 21 to *m/z* 300 (including five control parameters *m/z* 21, *m/z* 25, *m/z* 30, *m/z* 32, and *m/z* 37) were measured, with dwell time of 200 ms and for the time period of 24 h. Drift tube parameters included: pressure in range from 2.13 to 2.15 mbar; temperature 60 °C; voltage 600 V; E/N parameter 145 Td and reaction time 90 µs. The count rate of H_3_O^+^/H_2_O was 2.1 to 14.1% of the count rate of H_3_O^+^ ions, which was in the range 6.1 × 10^6^ to 9.7 × 10^6^ counts^−c^. Measurements were conducted on five independent plants. In order to avoid the possible deviations in experimental data caused by the changes in water content of plant tissue during rehydration, all results are normalized to the dry mass values (DW).

### 2.4. Determination of Relative Water Content

Plant material for measurements of relative water content (RWC) and LP was maintained in the same conditions as described for PTR–MS experiments and separately prepared from three independent plants each at different points of rehydration. RWC was determined in detached fronds at specific time intervals during rehydration process (every two hours) and calculated by using the equation.

RWC (%) = [(FW−DW)/(TW−DW)] × 100(1)

FW-fresh weight of fronds was measured immediately after sampling and then the fronds were left in distilled water for the next 24 h at 25 °C in the dark (TW-turgid weight). DW-dry weight was determined after lyophilization of fronds for 24 h (Lyovac GT2, SRK-Systemtechnik GmbH, Riedstadt, Germany).

### 2.5. Measurement of Lipid Peroxidation

LP was determined by MDA or thiobarbituric acid-reactive-substances (TBARS) assay as described by Hodges et al. [21] with some modifications. Briefly, 0.2g of fern fronds were homogenized in 5 mL of 80% ethanol and then centrifuged at 12,000× *g* for 20 min (Thermo Scientific Heraeus Biofuge Stratos, Heraeus Holding GmbH, Hanau, Germany). A 1-mL aliquot of sample extract was mixed with 1 mL of either (i)−TBA solution comprised of 20% (*w/v*) trichloroacetic acid and 0.01% butylated hydroxytoluene or (ii)+TBA solution containing the above plus 0.65% TBA. Samples were then mixed vigorously, heated at 95 °C in water bath for 25 min, and then quickly cooled on ice. After centrifugation at 12,000× *g* for 20 min, the absorbance of the supernatant was recorded at 440 nm, 532 nm and 660 nm (HP Agilent 8453 spectrophotometer, Agilent Technologies, Waldbronn, Germany). MDA equivalents were calculated by using the following equations.

[(A_532 + TBA_ − 3A_600 + TBA_) − (A_532 – TBA_ − A_600 − TBA_)] = A (2)

[(A_440 + TBA_−A_600 + TBA_) × 0.0571] = B (3)

MDA equivalents (nmol mL^−1^) = [(A − B)/157000] × 10^6^ (4)

Results were expressed as MDA equivalents per g of dry weight (nmolg^−1^ DW). Three biological replicates of each treatment were used for evaluation.

### 2.6. Statistical Analysis

Statistical analyses were performed by using STATGRAPHICS software, v. 4.2 (Statgraphics Technologies, Inc., The Plains, VA, USA). The data were subjected to the analysis of variance (ANOVA), and the comparisons between the mean values of treatments were made by the least significant difference (LSD) test calculated at the confidence level of *p* < 0.05. Linear regression analysis was performed; correlation coefficients (R) and coefficients of determination (R^2^) were calculated by using Statisticav. 10 software (StatSoft. Inc. 2011, Tulsa, OK, USA).

## 3. Results and Discussion

### 3.1. Relative Water Content Analysis

**Figure 1:**
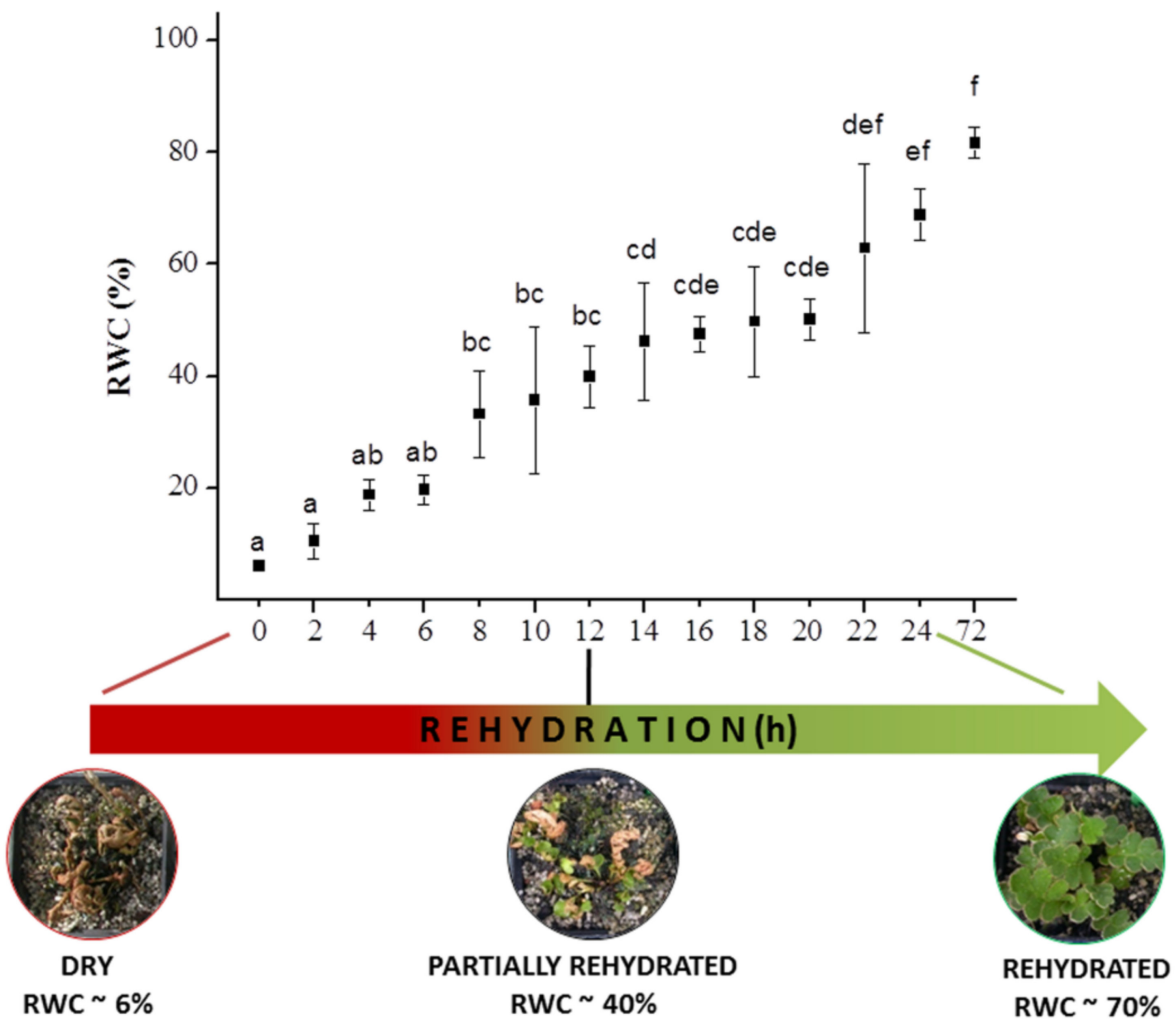
Rehydration response of rustyback fern (*A. ceterach*). Relative water content (RWC%) was determined during 72 h at the indicated time intervals and presented as the mean value of three independent measurements ± SE. Values with the same letter are not significantly different at the *p* < 0.05 level according to the LSD test. Three distinct phases of rehydration are shown at the bottom and correspond to dry plants at the beginning of experiment (0 h) and partly rehydrated (after 12 h) and rehydrated plants (24 h following rehydration).

Resurrection plants have the remarkable ability to survive extreme loss of water (desiccation) while staying dormant for a long period of time. Once the water becomes available again, the rehydration process starts and plants revive quickly and regains full metabolic activity in a few hours or days. In order to tolerate desiccation, resurrection plants must be able to limit the possible damage associated with drying in order to maintain physiological integrity during dehydration and to activate specific mechanisms upon rehydration in order to repair the damage caused during desiccation and subsequent rehydration [22]. Desiccation tolerant rustyback fern (*A. ceterach*) is capable of shifting from an active metabolic to anabiotic state, and vice versa, several times during its life cycle without permanent structural and/or functional damage [22]. Morphological changes in the form of frond folding and in-curling represent one of the main responses to desiccation stress. In the present study dormant sporophytes of rustyback fern (RWC 6%) were rehydrated in glass vessels until they regained full turgid state (RWC 82%) after 72 h of rehydration (Figure 1). The addition of water to desiccated ferns triggered the resurrection process and resulted in a gradual increase in RWC, accompanied by the unfolding of fronds. The rustyback fern sporophytes regained 40% of their RWC after 12 h of re-watering. The rehydration speed in the first 12 h was ~3% per hour and then the process slowed down slightly (between 12 h and 20 h of re-watering). One more boost in rehydration speed was observed in the last 4 h of the 24-h cycleand, afterwards, the process slowed down significantly (~0.3% per hour).

It should be noted that the recovery time of dry resurrection plants during rehydration varies widely, depending greatly on plant size and age, methods of rehydration, drying rate, desiccation extent, and duration before rehydration [23,24].Indeed, during the measurements of experimental parameters presented here, light conditions have changed with the day/night rhythm and, therefore, when the jars were illuminated, the temperatures inside were slightly higher than in the surroundings (up to 5 °C). Moreover, due to water evaporation and transpiration of the plants during the experiment, the humidity inside the jars was quite high (reaching almost 100%), which most probably affected the course of the experiment and behavior of the plants. Nevertheless, these light/temperature variations are in accordance with the frequent environmental changes in the natural habitat of *A. ceterach*, especially during the rainy season.

Similar results were obtained for resurrection plants from the family Gesneriaceae, such as *Haberlea rhodopensis* and/or species within the genus *Ramonda*, of which the latter often shares the same natural habitat with *A. ceterach* [25]. Slow water uptake during the initial hours of rehydration process could be considered as an adaptive defense mechanism for avoiding cellular damages by rapid water uptake upon rehydration [2]. Rakić et al. [26] stated that, at the beginning of rehydration, desiccation recovering plants go through an unstable and vulnerable short period, but then regain complete stability after 6 days of rehydration. Completely dried resurrection plants *Boea hygrometrica* restored their RWC very quickly upon rehydration, reaching 50% and 95% RWC within only 12 h and 24 h, respectively [27].

### 3.2. Emission of Volatiles during Rehydration

**Figure 2:**
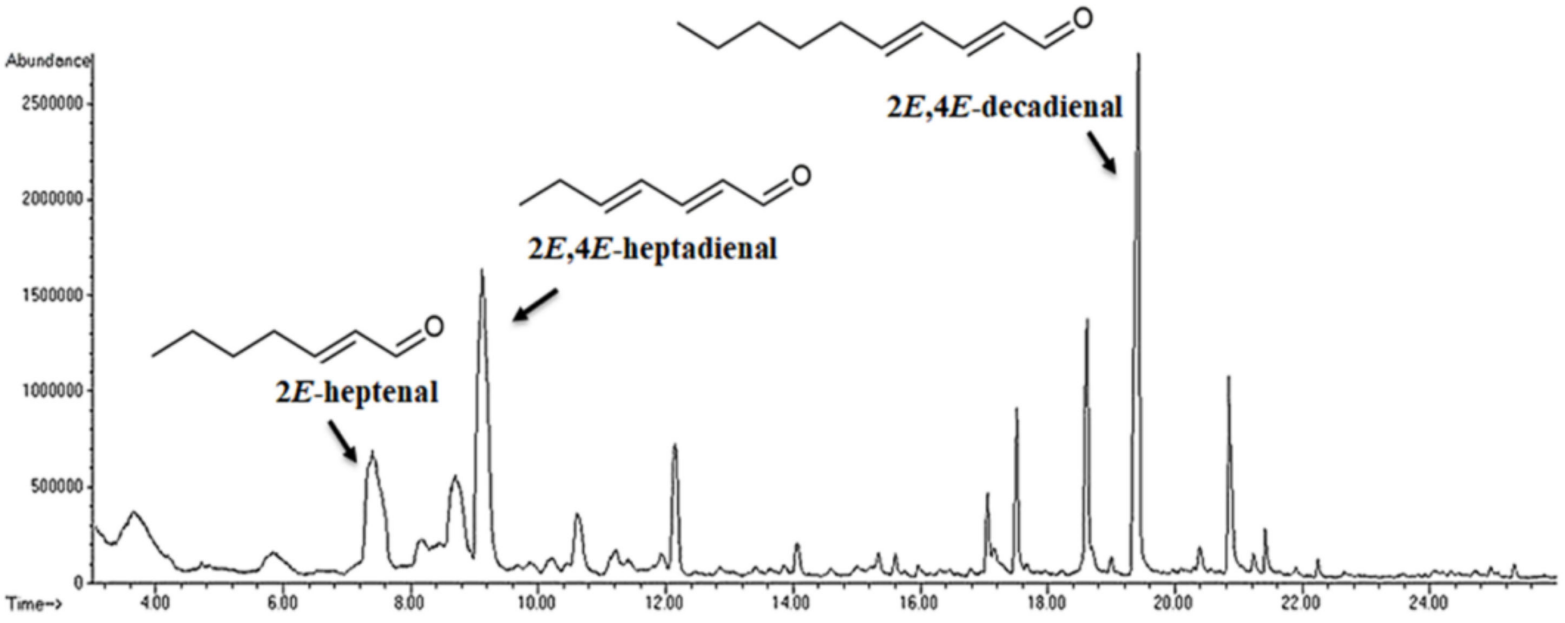
Headspace GC–MS chromatogram of VOCs detected in rustyback fern (*A. ceterach*). Peaks corresponding to the most abundant compounds are labeled.

Plants emit a wide variety of VOCs, mostly lipophilic compounds with low molecular weight, which could easily cross cellular membranes and be released into the surrounding environment [28]. According to their biosynthetic origin, VOCs can be divided into several major groups: terpenoids, phenylpropanoids/benzenoids, fatty acid derivatives and amino acid derivatives [29]. VOCs are engaged in plant growth and protection and their emissions are strongly dependent on the environmental conditions and developmental stages of the plant tissue. Plants exposed to abiotic and biotic stresses emit numerous compounds from almost all vegetative parts. Among the volatiles emitted from stressed plants, the enhancement of LOX pathway volatiles, which is a mixture of various C_6_ aldehydes and alcohols and their derivatives, represents atypical reaction. Many of these compounds are synthesized from the degradation of cellular structures and are used as indicators of cellular wall degradation or membrane denaturation [30]. The accessibility of Headspace GC–MS sampling together with PTR–MS analysis enabled research and examination of the changes in temporal and spatial emissions of VOCs [30,31,32]. Within the 40 compounds detected in rustyback fern during rehydration by Headspace GC–MS analysis, a pool of 30 volatiles was identified which represents 94.6% of the total VOCs composition (Figure 2, Table 1).

The volatile pattern of *A. ceterach* was dominated by isomeric heptadienals (>25%): (*E,E*)-2,4-heptadienal (17.66%) and (*E,Z*)-2,4-heptadienal (8.43%), followed by decadienals (>20%): (*E,E*)-2,4-decadienal (15.03%) and (*E,Z*)-2,4-decadienal (5.68%). Significant amounts of other fatty acid derivatives (alcohols and aldehydes) were also found, i.e., (2*E*)-heptenal (11.54%; intense green fatty odor), (*E*)-2-undecenal (4.51%; fruity waxy odor), 1-hexen-3-ol (4.48%; ethereal rum-like odor), n-nonanal (4.24%; hay odor), 3-octen-1-ol (3.05%; fatty fruity odor), (*E*)-2-decenal (2.81%; waxy fatty odor), 1-Octen-3-ol (2.37%; sweet, mushroom like odor), and n-heptanal (2.32%; bitter odor). On the other hand, aerial parts of rustyback fern showed a markedly lack of green odor C_6_-compounds, as well as monoterpene-type, sesquiterpene-type, and diterpene-type of hydrocarbons and/or corresponding terpenoids. Similarly, Froissard et al. [13] reported that VOCs profile of *A. ceterach* is dominated (77.4%) by lipid derivatives, mainly (*E*)-2-decenal, nonanal and (*E*)-2-heptenal, while significantly lower content of shikimic compounds (21.3%) and carotenoid derivatives (0.8%) was detected. The 1-octen-3-ol has also been reported from many mushrooms [33,34], ferns [35,36], and angiosperms [37]. (2*E*)-decenal, a natural plant and mushroom VOC with a plastic fatty odor, was also previously detected in some ferns (*Adiantum capillus veneris*, *Blechnum spicant*, and *Asplenium trichomanes*) [35,36]. In addition, (2*E*)-decenal was an abundant component of the strong “stink bug” scent together with (2*E,*4*Z*)-decadienal and (2*E,*4*E*)-decadienal [38]. These VOCs were also found in *Equisetum ramosissimum* and *E. scirpioides* together with the (2*E*)-heptenal, while (2*E,*4*Z*)-heptadienal and (2*E,*4*E*)-heptadienal were detected in *E. scirpioides* and *E. hyemale* [39]. It has been reported that n-heptanal, 2,4-decadienal, and 2,4-heptadienal exhibit fishy odors [40] and represent the main metabolic products of some algal species [41].

2,4-decadienal has been known, for a long time, as a product of deteriorated fat compounds [42] and as a widespread volatile constituent of dry fruits [43]. Similar compounds are produced by higher flowering plants and are believed to have a significant role in plant defense by acting as chemical attractants, alarm signals against herbivore attack, and/or protective compounds [44]. In addition, these polyunsaturated aldehydes have been reported to interfere with the reproductive success of some marine invertebrates [45]. One must bear in mind that 2,4-unsaturated aldehydes are chemically highly reactive. The generation of 2,4-decadienal represents the fast response of cells to the changes in their membrane composition and, unlike other signaling compounds (ethylene, superoxide, jasmonic acid, salicylic acid, etc.), does not require a preceding activation of gene expression. Therefore, the products of the oxidative membrane LP constitute “biological signals”, which produce nonspecific responses to a large variety of environmental stresses [46].

PTR–MS has become a commonly utilized technique for the analysis of trace amounts of VOCs and it offers many advantages over other conventional analytical methods. This is an online and non-invasive method with high sensitivity (parts per trillion/parts per billion concentrations) for plant VOCs assessment in real time at high throughput [31,47,48]. The main drawback of this technique is related to providing information limited to protonated molecular mass, which is not a specific indicator of chemical identity. Furthermore, the identification of the compounds is further complicated by the overlapping spectra of different VOCs species. In this study a chromatographic step (Headspace GC–MS characterization of volatiles in the atmosphere of glass jars), enabled the selection and identification of specific VOCs in rustyback fern fronds, and subsequently their dynamics during the process of rehydration was monitored by PTR–MS. PTR–MS and GC–MS, as complementary techniques for the analysis of volatiles which enable rapid quantification of selected substances [32].

**Scheme 1:**
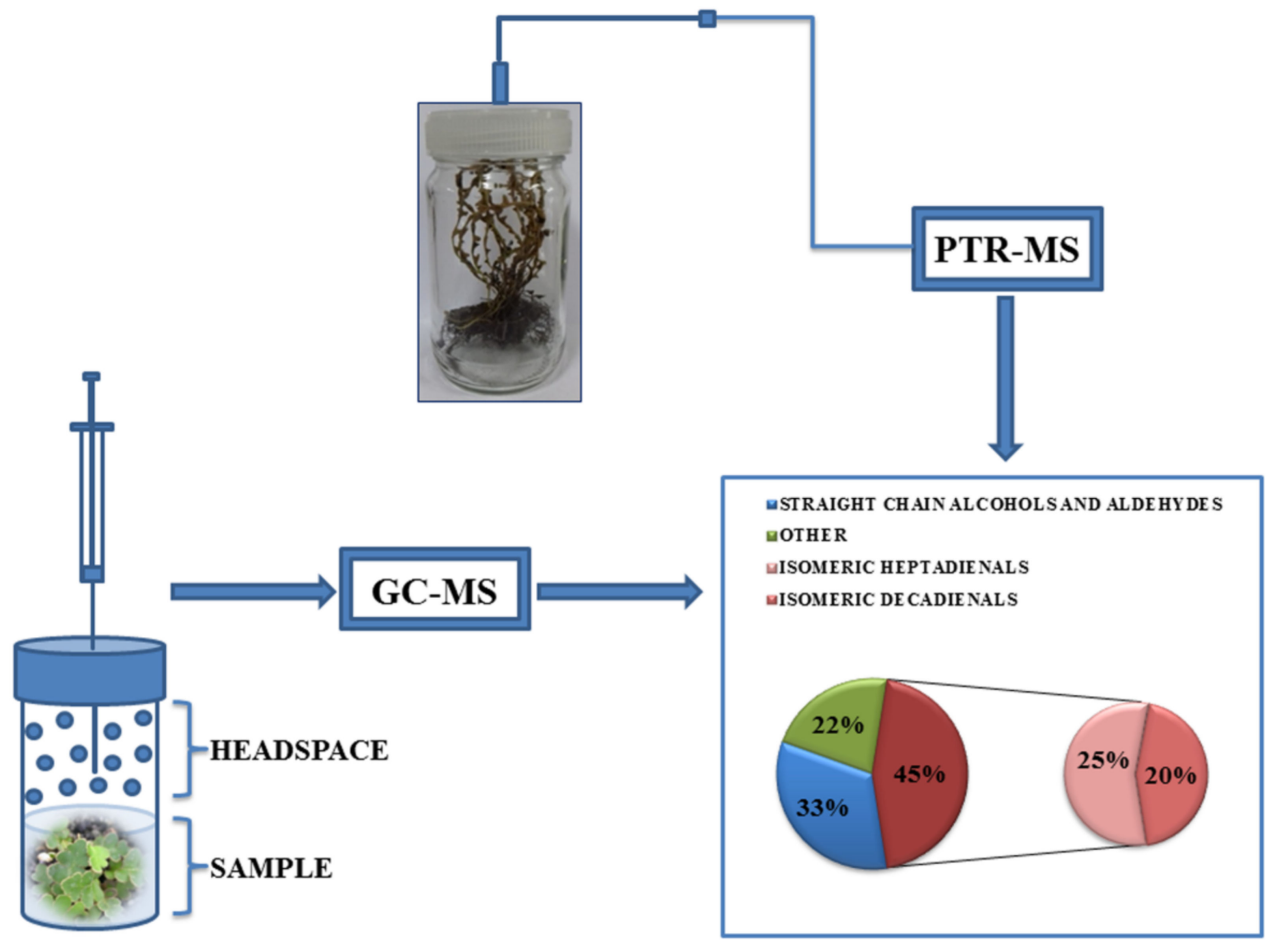
A number of peaks corresponding to the protonated masses of the individual VOCs may be observed, including a smaller contribution of VOCs’ fragments and clusters. Nonetheless, based on the GC–MS analysis, peaks which correspond to *m/z* 41, 43, 53, 70, 81, 83, 110 and 128 were of main interest.

PTR–MS real-time detection and analysis of VOCs emission from the rehydrating rustyback fern sporophytes was performed by using desiccated (dormant) plants. The measurements started with the initiation of the rehydration process; that is, when the water was added to the plants (Scheme 1).

**Figure 3:**
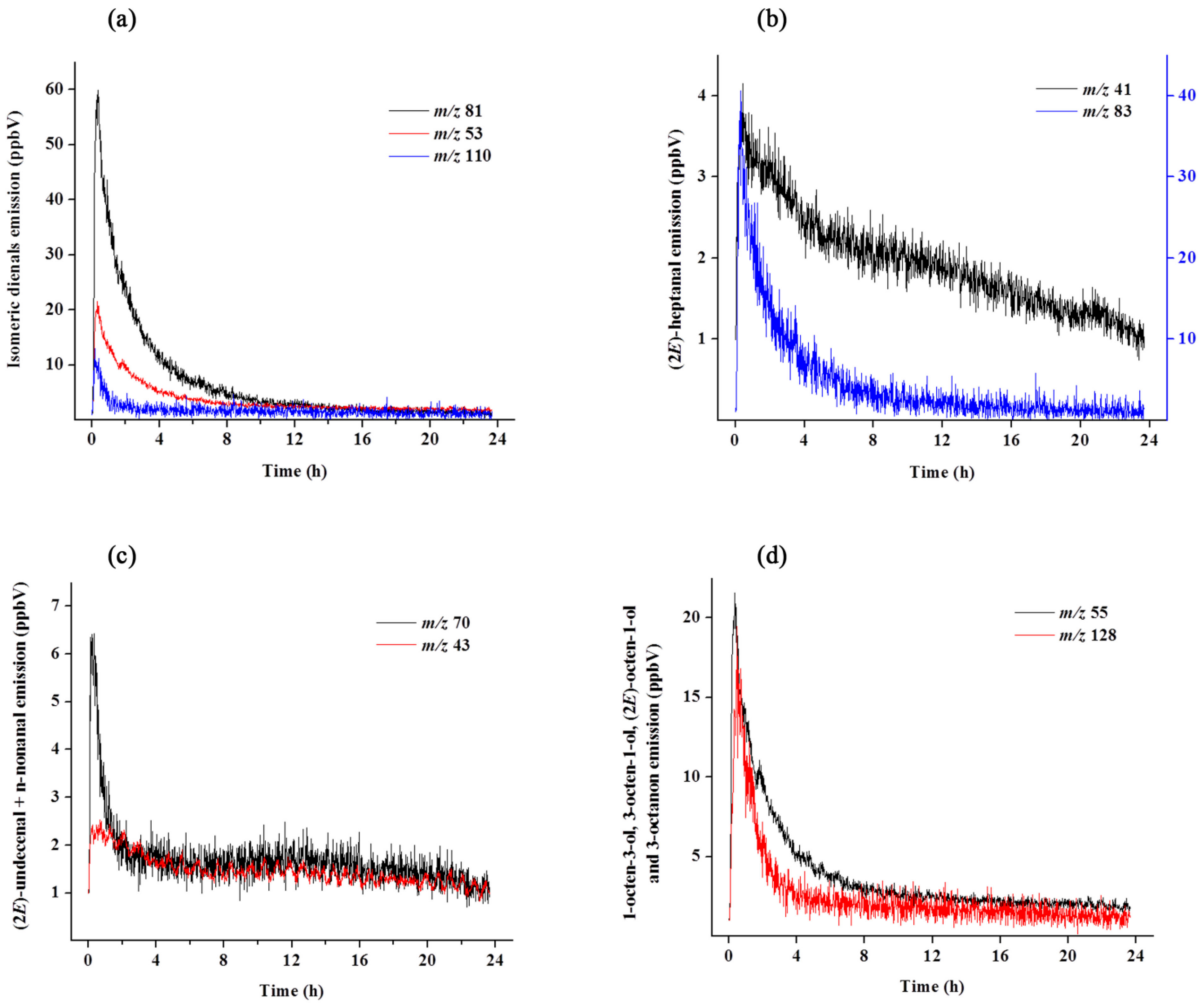
Time course of VOCs emission from rustyback fern (*A. ceterach*) fronds during the process of rehydration detected on-line by the PTR–MS method. Different colors indicate different ions corresponding to distinct volatiles: (**a**) Isomeric dienals, *m/z* 81 + *m/z* 53 + *m/z* 110; (**b**) (2*E*)-heptenal, *m/z* 83 + *m/z* 41; (**c**) (2*E*)-undecenal + n-nonanal, *m/z* 70 + *m/z* 43; (**d**) 1-octen-3-ol + 3-octen-1-ol + (2*E*)-octen-1-ol + 3-octanon, *m/z* 55 + *m/z* 128. Data shown represent mean sequence of plants from five independent experiments normalized to DW values.

PTR–MS online detection of VOCs (as was previously shown by Headspace GC–MS analysis) revealed that the rehydration process enhanced the isomeric dienals emission rates. There was a striking signal at *m/z* 81 that was assigned to both 2,4-heptadienals and 2,4-dicadienals, which was apparently elevated at the onset of rehydration process, reaching 59.88 ppbV during the first hour and then declining progressively with the further increments of RWC (Figure 3a). The emission intensity of other fragments belonging to the isomeric alkadienals, with *m/z* 53 and *m/z* 110, followed the same trend and reached 21.58 or 13.01 ppbV, respectively. Furthermore, intensity levels of selected fragments were significantly correlated with *m/z* 81 emission (R^2^ = 0.99588 and R^2^ = 0.7781, respectively). Approximately 12 h after the onset of experiments, when the recorded RWC of the plant tissue was 40% (Figure 1), the concentration of isomeric dienals decreased and returned to the basal levels. Similar trends were also observed for the other targeted compounds.

The signals at *m/z* 83 and *m/z* 41 were attributed to 2*E*-heptenal, which belongs to the group of monounsaturated fatty aldehydes. The amount of compound with signal at *m/z* 83 emitted by rustyback fern fronds was increasing over the rehydration process with a correlation between distinct fragments of R^2^ = 0.81391. The highest intensity of the *m/z* 83 signal was detected within the first hour of rehydration (up to 40.57 ppbV), with an almost complete absence of the signal 12 h after the start of measurement. Still, *m/z* 41 fragment showed a 10-fold lower emission rate (4.15 ppbV) compared to *m/z* 83, but remained significantly abundant towards the end of the experiment (Figure 3b). Emission of *m/z* 70 (6.44 ppbV) and *m/z* 43 (2.54 ppbV) was attributed to (2*E*)-undecenal and saturated fatty aldehyde n-nonanal, respectively (Figure 3c).

A wide variety of VOCs with the Mr 128 was recorded in rustyback fern sporophytes during the rehydration process, such as 1-octen-3-ol, 3-octen-1-ol, (2*E*)-octen-1-ol, and 3-octanon. Emissions of *m/z* 128 and *m/z* 55, selected as fragments present in all listed compounds, were 19.48 ppbV and 21.58 ppbV, respectively, with the significant correlation value of R^2^ = 0.93138 (Figure 3d). The emission levels of ions corresponding to all the above mentioned compounds were increasing over the experimental time, with the maximum values reached during the first two hours of re-watering, as has already been outlined for other detected volatiles. However, the possible contribution of hydronium water cluster H_3_O^+^ (H_2_O)_2_ to the *m/z* 55 fragment intensity should not be neglected [49].

(*E*)-2-heptenal is a well-known and important volatile aldehyde formed during linoleic acid oxidation. Lee and Min [50] reported that, among other compounds, (*E*)-2-heptenal, 1-octen-3-ol, 2,4-heptadienal, 2-octen-1-ol, 2,4-octadienal, and 2,4-decadienal were identified as volatile oxidation compounds (VOOs) in chlorophyll mixed with linoleic acid under light storage. In addition, (*E*)-2-heptenal was detected as the main VOOs in olive oil exposed to light irradiation [51], while grape, flax, and black cumin seed oils had high content of (*E*)-2-heptenal, 2,4-hexadienal, and 2,4-heptadienal throughout the photo-oxidation conditions [52]. (2*E*)-undecenal occurs naturally in coriander leaves [53] and red pepper fruits [54]. Nonanal could be found in a large quantity in different plant species, such as tomato, canola, soybean, etc., and it has been reported to show antimicrobial activity against various bacterial and fungal pathogens [55]. Nonanal, together with 1-hexanal, *cis*-3-hexenol, and methyl jasmonate, belongs to the fatty acid derivative class of plant VOCs, which arise from C_18_ unsaturated fatty acids, linoleic or linolenic [29].1-octen-3-ol was mostly found to be released by pathogenic or endophytic fungi [56]. However, recent studies have shown that this compound is also emitted from a number of legume species [57]. Consequently, nonanal, 3-octanone, and 1-octen-3-ol were designated as main compounds responsible for the off-odor of infected strawberry fruit [58].

### 3.3. Estimation of Lipid Peroxidase Activity

**Figure 4:**
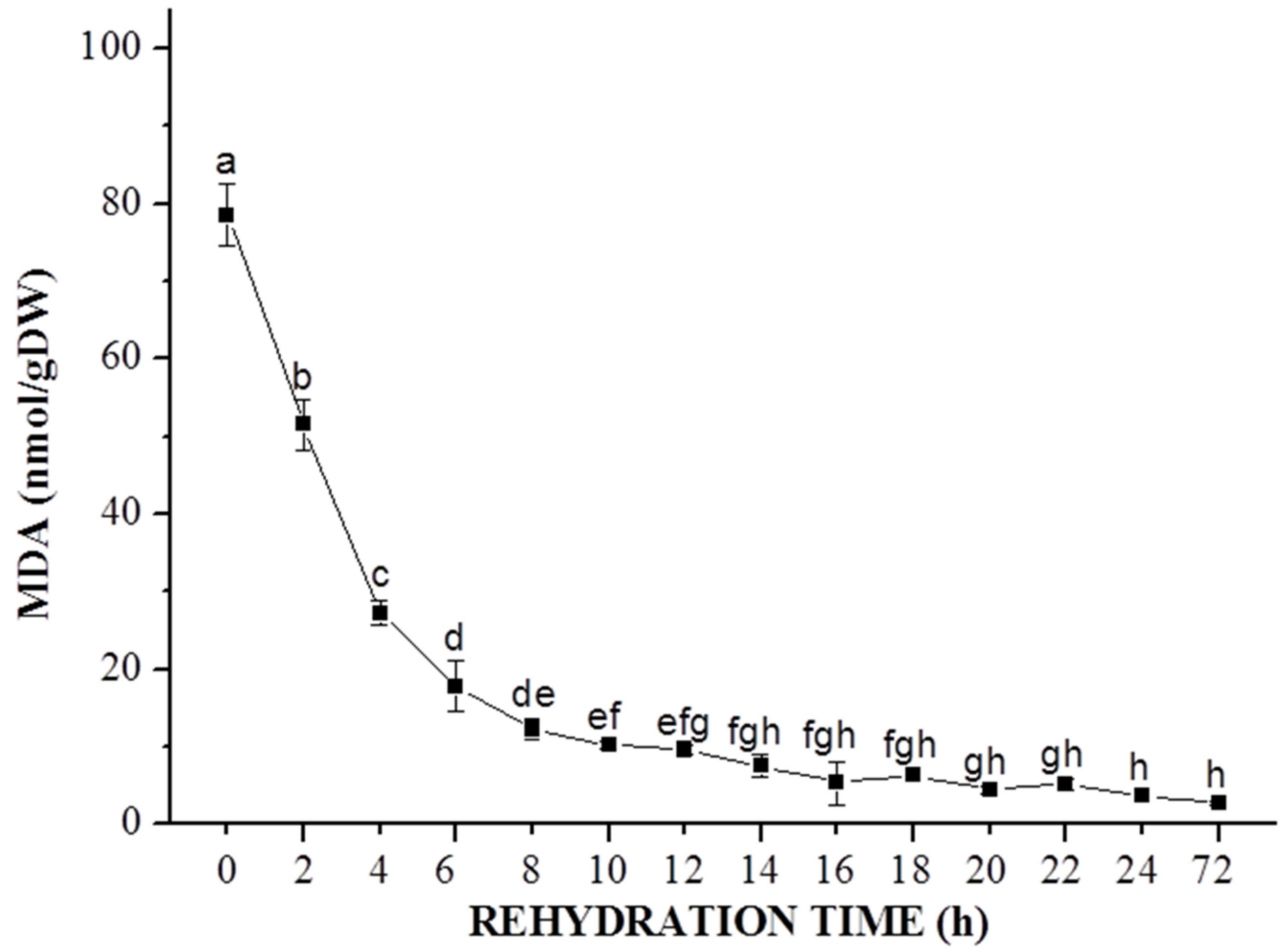
Determination of lipid peroxidation in fronds of rustyback fern (*A. ceterach*) during the process of rehydration. Results of MDA assay are presented as the means of three independent measurements ± SE. Values with the same letter are not significantly different at the *p* < 0.05 level according to the LSD test.

The determination of membrane lipid peroxidation is often based on the measurements of MDA, as a product of unsaturated fatty acid peroxidation thatis commonly used as a biomarker of oxidative lipid injuries [59,60]. Lower MDA content indicates less oxidative damage, and was described as an indicator of more efficient stress tolerance. LP level, expressed as MDA content, in desiccated fronds of *A. ceterach* was ~80 nmol g^−1^ DW and sharply declined during the initial six hours of rehydration (Figure 4). Between 8 h and 24 h of rehydration, the MDA level gradually decreased to ~5 nmolg^−1^ DW, reaching the control values.

Desiccation stress in plants can result in lipid destruction and membrane damage due to free radical production. In plants under abiotic stress (e.g., drought), the enhanced formation of ROS was noted, which could easily oxidize PUFAs. PUFAs are lipid components in plant membranes, and their reaction with ROS triggers serial LP [59]. Peroxidation of lipid membrane is considered as the main damaging effect of ROS. Since plant cell structures rich in PUFAs (cell plasma membranes and chloroplast) are also exposed to the generation of ROS, there is a high risk of LP in these plant organelles. Higher MDA levels at the beginning of rehydration could indicate that oxidative damage of *A. ceterach* fronds has occurred during desiccation process to some extent. Niinemets et al. [61] reported the emission burst of LOX volatiles upon re-watering in desiccation tolerant epiphytic filmy ferns from the family Hymenophyllaceae, suggesting a fast enhancement of ROS production. Similarly, LP level, expressed in terms of lipid hydroperoxide (LOOH) content, increases as the fronds of resurrection fern *Pleopeltis polypodioides* dehydrates, and rapidly decreases after rehydration [62]. The decrease in lipid peroxidation during rehydration was detected in fronds of *Selaginella brachystachya* [63], *S. tamariscina* [64] and *S. bryopteris* [65]. Similar patterns of change of LP were also revealed during the recovery of angiosperm *Haberlea rhodopensis* [66]. Conversely, in angiosperm *Paraisometrum mileense*, MDA levels were maintained close to the control values in both completely dehydrated and well rehydrated leaves [67].

Results from studies on desiccation tolerance mechanism in resurrection fern *Adiantum raddianum* indicated that significant damage was induced on membrane integrity due to desiccation stress [68]. Furthermore, desiccation appeared to cause some damage in the cellular membranes of *Adiantum latifolium*, but this process was reversed and/or repaired upon rehydration to control values, suggesting that the antioxidant system was efficient in scavenging ROS, thus helping the plant to recover from the oxidative stress [69]. These findings, together with our results, support the thesis that much stronger volatile responses might be expected in homoiochlorophyllous species that maintain pigments and photosynthetic activity through desiccation. Although elevated MDA level in desiccated fronds of *A. ceterach* might indicate the oxidative degradation of PUFA residues of the membrane lipids and putative disturbed membrane integrity, we can speculate that repair mechanisms of this resurrection fern permitted reversible changes in the peroxidation of the membrane lipids and the rapid regaining of membrane configuration upon rehydration. Our results demonstrate the complete recovery of *A. ceterach* sporophytes after losing more than 90% of cellular water content.

Relatively high production of VOCs that are potentially associated with the considerable amounts of polyunsaturated aldehydes in rustyback fern sporophytes could have significant implications from biotechnological and ecological perspectives, since these plants could be taken into account as a valuable source for these bioactive compounds. Further research on the lipid metabolism in rustyback fern would provide better insight into the mechanisms that protect the integrity of membrane lipids of the cell, including thylakoid lipids. Considering that restoration of vital functions and metabolic activities represent an integral part of the entire phenomenon of desiccation tolerance, this could highlight the importance of lipids and membrane-protecting compounds in *A. ceterach* during dehydration/rehydration process, and it will be the main focus of our future research.

## 4. Conclusions

Desiccation tolerant rustyback fern *(Asplenium ceterach*) revive upon watering and restores physiological activity within the 72 h. Rehydration was characterized with different metabolic changes which led to the complete recovery from a dry state, including increased emission of volatiles mainly belonging to the fatty acid derivatives. Lipoxygenase activity was not stimulated during the rehydration according to the decreased level of MDA as an indicator of membrane damage. Further research on lipid metabolism in rustyback fern would provide better insight into the mechanisms that protect the integrity of the cell membranes during dehydration/rehydration cycle.

## Figures and Tables

**Table 1:** Volatile organic compounds (VOCs) composition of rustyback fern (*A. ceterach*) as revealed by Headspace GC–MS analysis.

No.	Constituent	KIE	KIL	*t*_R_	% (m/m) ^1^
1	Hex-1-ene-3-ol	829.5	832	3.66	4.48
2	n-Heptanal	903.8	901	5.88	2.32
3	(2*E*)-Heptenal	954.6	947	7.40	11.54
4	1-Octen-3-ol	980.0	974	8.16	2.37
5	3-Octanone	985.1	979	8.31	0.90
6	2-Pentyl furan	988.8	984	8.42	1.13
7	(2*E*,4*Z*)-Heptadienal	1000.1	988	8.76	8.43
8	(2*E*,4*E*)-Heptadienal	1010.7	1005	9.13	17.66
9	(2*E*)-Octen-1-al	1043.1	1034	10.23	0.96
10	Oct-3-ene-1-ol	1054.2	1044	10.61	3.05
11	n-Octanol	1071.2	1063	11.19	1.32
12	(2*E*)-Octen-1-ol	1078.5	1067	11.44	0.71
13	n-Undecane	1092.4	1100	11.93	1.40
14	n-Nonanal	1099.1	1100	12.15	4.24
15	(2*E*)-Nonen-1-al	1154.2	1157	14.07	0.89
16	n-Dodecane	1190.9	1200	15.35	0.58
17	n-Decanal	1198.3	1201	15.61	0.41
18	g-Octalactone	1241.2	1250	17.05	1.55
19	n.i.	1244.2	/	17.15	1.06
20	(2*E*)-Decenal	1254.8	1260	17.51	2.81
21	(2*E*,4*Z*)-Decadienal	1287.4	1292	18.62	5.68
22	n-Undecanal	1299.0	1305	19.00	0.42
23	(2*E*,4*E*)-Decadienal	1311.6	1315	19.42	15.03
24	n.i.	1342.1	/	20.40	0.70
25	(2*E*)-Undecenal	1355.8	1357	20.85	4.51
26	n.i.	1367.8	/	21.23	0.39
27	n.i.	1373.3	/	21.41	0.79
28	n-Dodecanal	1398.8	1408	22.24	0.30
29	n-Tridecanal	1499.5	1509	25.33	0.21
30	(6*Z*)-Pentadecen-2-one	1655.5	1667	29.79	0.16
31	(2*E*)-Tridecenol acetate	1701.1	1703	31.07	0.18
32	Hexadecanoic acid	1965.8	1959	37.75	0.30
33	n.i.	2062.5	/	40.02	0.23
34	Linoleic acid	2146.9	2132	41.89	0.77
35	n.i.	2153.2	/	42.02	0.32
36	n.i.	2282.9	/	44.79	0.28
37	n.i.	2455.6	/	48.25	0.43
38	n.i.	2460.5	/	48.35	0.66
39	Squalene	2808.6	2814	54.74	0.31
40	n.i.	3062.7	/	59.96	0.52
	Number of detected constituents			40	100%
	Number of identified constituents			30	94.61%

^1^ KIE—Kovats (retention) index experimentally determined (AMDIS, uncorrected); KIL—Kovats (retention) index—literature data [17]; *t*_R—_retention time (min); n.i.—not identified; % (m/m)—percentage of component emitted (mass on mass).

## Data Availability

The data presented in this study are available upon request from the corresponding authors.
